# Longitudinal analysis of HIV-risk behaviors of participants in a randomized trial of prison-initiated buprenorphine

**DOI:** 10.1186/s13722-019-0172-2

**Published:** 2019-12-02

**Authors:** Thomas R. Blue, Michael S. Gordon, Robert P. Schwartz, Kathryn Couvillion, Frank J. Vocci, Terrence T. Fitzgerald, Kevin E. O’Grady

**Affiliations:** 1 Friends Research Institute Inc., 1040 Park Avenue, Suite 103, Baltimore, MD 21201 USA; 2 Glenwood Life Counseling Center, 516 Glenwood Avenue, Baltimore, MD 21212 USA; 3 Department of Psychology, University of Maryland, College Park, 4094 Campus Drive, College Park, MD 20742 USA

**Keywords:** Hiv-risk behavior, Buprenorphine, Prison, Opioid use disorder, Correctional settings

## Abstract

**Background:**

It has been estimated that approximately 15% of people who are incarcerated in the US have histories of opioid use disorder. Relapse to opioid use after release from prison poses a serious risk of HIV infection. Prison-initiated buprenorphine may help to reduce HIV infection given the association between opioid use and HIV-risk behaviors.

**Methods:**

The present study is a secondary analysis of longitudinal data gathered from a randomized controlled trial of buprenorphine-naloxone for people who were incarcerated (*N* = 211) between 2008 and 2012. It compares the impact of assignment to initiate buprenorphine in prison (*N* = 106 randomized, *N* = 104 analyzed) versus in the community (*N* = 107 randomized, *N* = 107 analyzed) and whether or not participants entered community treatment on the frequency of HIV-risk behaviors in the 12 months following release from prison. Data were analyzed hierarchically and for each outcome variable, a multilevel, over-dispersed Poisson model was fit to the data. Outcome variables were the number of times the following behaviors occurred in the last 30 days: (1) having sex without a condom (2) injecting drugs (3) using unsterilized needles, and (4) sharing injection paraphernalia.

**Results:**

Participants assigned to begin buprenorphine in the community experienced a greater decrease in injection drug use over time compared to participants assigned to begin buprenorphine in prison. There were no significant associations between treatment assignment or community treatment entry and instances of having sex without a condom, sharing injection paraphernalia, or using unsterilized needles.

**Conclusions:**

Overall, the present study did not find support for the initiation of buprenorphine in prison (as opposed to the community) as a means to reduce incidences of HIV-risk behaviors. Avenues for future research in the nexus of HIV-risk reduction, criminal justice, and pharmacotherapy are discussed.

*Trial registration *This study was supported by the National Institute on Drug Abuse (NIDA), *Buprenorphine for Prisoners* (PI: Kinlock; R01DA021579). ClinicalTrials.gov identifier: NCT 00574067

## Background

The United States (US) leads the world in both the number of incarcerated persons and rate of incarceration. Figures from 2016 estimate the total number of incarcerated persons in the US at 1.5 million and a rate of incarceration in jails or prisons of 670 per 100,000 [1]. Compared to the general population, incarcerated persons have a disproportionally higher rate of opioid use disorders (OUDs) [2–4]; 13.1% and 9.2% of people incarcerated in state and federal prisoners respectively reported using heroin or other opiates regularly in the community before their incarceration [5]. Given that the vast majority of incarcerated persons will be released from prison at some point and that 626,024 individuals were released from state and federal prisons in 2016 [6], there is a considerable need to deliver effective treatment to this population in order to reduce relapse to drug use upon release.

A return to illicit psychoactive substance use upon community re-entry poses significant risks to health and public safety [7]. Individuals recently released from jail or prison are at increased risk for overdose death within their first month in the community [8–17] and a return to opioid use is associated with criminal activity [7, 18, 19] and re-incarceration [18, 20, 21]. Relapse to drug use is also a significant public health concern as it heightens the risk for HIV and hepatitis B and C infections [2, 4, 7].

The Centers for Disease Control and Prevention (CDC) estimated that in the US there are approximately 1.1 million people who were HIV positive in 2015 [22]. Of that group, one in seven individuals were unaware of their HIV status [22]. In 2015, HIV was the 9th leading cause of death for those 25 to 44 years of age [22]. As with OUDs, HIV infection is overrepresented in the criminal justice population with estimated rates ranging between three and five times greater than that of the general population [23–25]. The higher incidence of HIV infection amongst criminal justice populations is at least partially attributable to their increased rates of substance use disorders. Substance use exacerbates the risk of HIV through the practice of injection drug use, the sharing of needles and injection paraphernalia (cookers, cotton, and rinse water), and its association with risky sex, sex with multiple partners, and transactional sex/sex work [26–30]. For example, a longitudinal study by MacGowan and colleagues found that among men recently released from prison, the only factor independently associated with risky sex was the use of alcohol or illicit substances before sex [31]. Substantial research evidence indicates that men and women with OUDs differ in terms of their health and substance use treatment needs and their risk of HIV infection. Among individuals with OUDs, women are more likely than men to suffer from serious medical conditions [32–34], mental health problems [33, 35], unemployment [35, 36], the stress of having responsibility for child care [37], and the burden of having a spouse or partner with addiction problems [33, 34]. Moreover, an analysis by Binswanger and colleagues [38] of HIV risk behaviors for both men and women post-release found that a higher proportion of women than men engaged in several risk behaviors including unprotected sex and sex with multiple partners. In addition, they found that women were more prone than men to exchange sex for drugs and/or money.

While the practice of mandatory or opt-out HIV testing has become more commonplace in state and federal prisons, [25] there is still a need for HIV risk-reduction interventions for criminal justice involved individuals [26]. One promising avenue for improving public health outcomes, is the use of opioid agonist therapy (OAT) for the treatment of OUD. Reviews of studies involving OAT and HIV-risk behaviors have shown that both methadone and buprenorphine may help to reduce injection drug use, needle sharing, and risky sexual behaviors [26, 39]. However, these studies often focus on community samples and there is limited research on the effects of prison-initiated OAT on HIV-risk behaviors [39, 40] presenting an opportunity for study.

For the treatment of OUDs, the three pharmacotherapies approved by the U.S. Food and Drug Administration (methadone, buprenorphine, and naltrexone) represent the highest standard of care, but are rarely implemented within the criminal justice system [41–43]. Given their demonstrated efficacy in community settings [44, 45], implementation of pharmacotherapies prior to release may help to prevent illicit opioid use in prison and relapse upon release for individuals who have maintained opioid abstinence while incarcerated. In turn, a reduction in the rates of relapse to opioid use upon release can help to reduce the incidence of HIV infections through a reduction in injection drug use and risky sex behaviors. A review of studies of pre-release opioid agonist therapies (OAT) found that pre-release OAT in prison is associated with significantly increased treatment uptake after release and treatment retention, with differences observed as far as 12 months post-release [46]. Given the importance of substance use treatment retention for positive treatment outcomes [47, 48], this finding supports the use of pre-release OAT for reducing relapse to opioid use and its associated harms, including the risk of HIV infection.

Our research group has previously reported on findings from a randomized clinical trial that compared post-release outcomes of incarcerated persons with pre-incarceration histories of opioid dependence (defined by DSM-IV) who were randomly assigned to begin sublingual buprenorphine/naloxone prior to versus post-release from prison [49, 50]. The study found that participants who were randomly assigned to initiate buprenorphine in prison were significantly more likely to enter and to remain in buprenorphine treatment in the community compared to participants who were assigned to begin buprenorphine treatment after release [50]. However, despite greater community treatment exposure, there were no significant differences between treatment conditions in heroin and cocaine use at the 12-month follow-up [50].

### The present study

The aim of the present study is to examine the impact of initiating buprenorphine prior to versus post-release on relative incidences of four key HIV risk behaviors: (1) sex without a condom (2) injection drug use (3) using unsterilized needles, and (4) sharing injection paraphernalia. Here we present findings from a secondary analysis of data from the above-mentioned clinical trial. We hypothesized that because of the potential advantages of initiating buprenorphine treatment in prison (higher rates of treatment entry and treatment retention) that there would be greater levels of improvement (greater decreases) in the number of self-reported incidences of each of the four HIV risk behaviors over time when controlling for gender and community treatment entry. The parent study found that the study condition assigned to initiate buprenorphine in prison compared to after release was associated with significantly higher rates of community treatment entry and community treatment exposure. While greater participation in treatment post-release did not produce significant differences in heroin or cocaine use, it may have impacted other aspects of substance use that are related to increased HIV risk (i.e. using unsterilized needles, exchanging sex for drugs) especially when treatment is based on the principle of harm reduction.

## Methods

### Parent study

Participants in the parent study were 211 adults incarcerated in prison with a history of DSM-IV defined opioid dependence in the year prior to their index incarceration. They were recruited between 2008 and 2012 within 3 to 9 months prior to their release. All participants regardless of condition assignment were offered 12 weekly group-based substance use counseling sessions in prison. They were randomly assigned to initiate sublingual buprenorphine/naloxone, hereafter termed buprenorphine, either in prison (*N* = 106 randomized, *N* = 104 analyzed, *N* = 2 missing all data) or post-release (*N* = 107 randomized, *N* = 107 analyzed). Additionally, they were randomly assigned to receive buprenorphine treatment post-release in either an opioid treatment program (OTP) or in a community health center (CHC). In partnership with the participating community treatment facilities, participants in the study were granted guaranteed admission if they reported to their assigned facility within 10 days of release. However, because the parent study found no differences between assignment following release into the community to an OTP or a CHC, the present study focuses on the condition assignment to initiate buprenorphine either pre- or post-release from prison. Buprenorphine was started at low doses (1/0.25 mg buprenorphine/naloxone daily) and increased slowly (e.g., increase of 1/0.25 mg per week until reaching 4/1 mg with subsequent increases by 2/0.5 mg per week to reach 8/2 mg) because most participants were not opioid tolerant at the time of study recruitment. This dosing schedule is much slower than would be used for opioid-tolerant patients in the community. Vocci et al. [51] provide a detailed treatment of buprenorphine dose induction for non-opioid tolerant pre-release incarcerated persons. Prior to release, exit interviews were held with participants where the importance of promptly reporting to their designated post-release treatment facility was emphasized. Participants were also given business cards containing information about the community-based treatment program they were assigned to attend [52]. Eighty-two (38.9%) of participants entered community treatment within 10 days. A detailed description of the methods and outcomes of the parent study can be found elsewhere [49, 50, 52]. The parent study was approved by the Friends Research Institute’s Institutional Review Board, the Maryland Department of Public Safety and Correctional Services Research Committee, and the Federal Office for Human Research Protections. The ClinicalTrials.gov identifier is NCT 00574067.

### Measures

#### Predictor variables

The predictor variables can be considered as either treatment or control variables. The treatment variable, the main variable of interest, was the assigned treatment condition: buprenorphine initiated in prison versus buprenorphine initiated in the community. The control variables were participant community treatment entry and gender. Participants were considered to have successfully entered community treatment if they reported to their assigned buprenorphine treatment program within 10 days of release. The control variables are included in the analyses so that the effect of treatment condition can be ascertained above and beyond the effects of community treatment entry and participant gender.

#### Outcome variables

As part of the study protocol, HIV-risk behaviors were reported at study entry (in prison) and at 1, 3, 6, and 12 months post-release using the Texas Christian University HIV/AIDS Risk Assessment. This measure assesses HIV knowledge and sexual and injected-related risk behaviors during the preceding 30-day period. It has been used to assess the effectiveness of community outreach interventions on reducing AIDS risk [53] and to study the cognitive and psychosocial factors associated with HIV/AIDS risk behavior [54, 55]. Data were in the form of self-reported number of times engaging in each of the following behaviors in the last 30 days: (1) having sex without a condom (2) injecting drugs with a needle (3) using unsterilized (“dirty”) needles, and (4) sharing injection paraphernalia (“works”). In the case of self-report data at study entry (during their index incarceration), participants recalled the number of times engaging in each behavior during their last 30 days in the community. Although follow-up measures were scheduled to be collected at specific, fixed times during the post-release period, there were sometimes significant variations among participants in the actual dates at which assessments were completed or data for the outcomes of interest were missing at some follow-up intervals (rate of missing responses: 54%) due to the numerous challenges of gathering longitudinal data from criminal justice-involved adults [56] including a lack of access to reliable transportation, unstable housing, requirements of community supervision, and re-arrest and re-incarceration [57, 58]. Therefore, the point in time when each assessment was collected was scaled as the number of days since the baseline assessment. Because each of the four outcomes were evaluated by open-response items, participants were free to enter any number for the incidences of each risk behavior that occurred in the past 30 days. In some cases, the self-reported incidences of HIV-risk behaviors appear to have been unrealistically large (e.g., injecting drugs 900 times in 30 days). To prevent these responses from distorting the results, a datum that exceeded 300 self-reported incidences of behavior in the last 30 days (indicating an average greater than 10 times per day) was treated as missing, an event that occurred in 12 instances. Given the flexibility of hierarchical linear models to deal with missing data, all other responses from participants with deleted data were retained. In total, over 2000 responses were collected during the 12-month follow-up period meaning that deleted data represented less than 0.01% of all responses.

### Statistical analysis

**Fig. 1:**
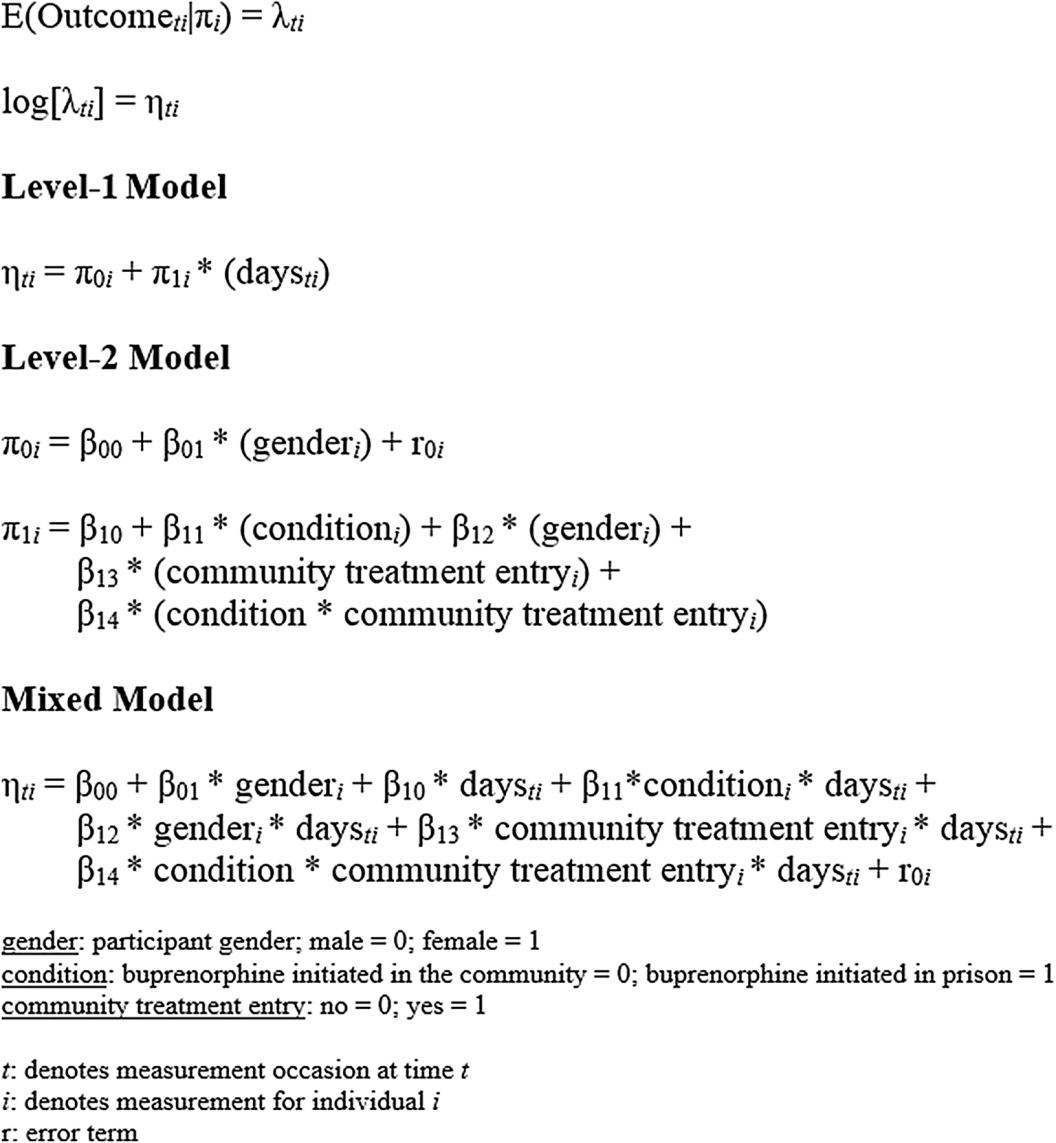
Equations for the multilevel, over-dispersed Poisson models fit to each of the four outcome measures

Data were evaluated longitudinally in order to account for the effects of time and to examine how the treatment and control variables were related to the rate of change in HIV-risk behaviors over time. In order to accomplish this goal, a hierarchical framework was adopted. Observations at separate time points (level-1 data) were considered nested within participants (level-2 data). A hierarchical framework is useful for the evaluation of repeated measures data because of its flexibility to accommodate unbalanced data structures where the data for some (or all) individuals is incomplete or when participants are measured at different sets of time points [59]. In order to achieve the aim of the present study, a multilevel, over-dispersed Poisson model was fit to the data for each of the four outcomes: (1) sex without a condom (2) injection drug use (3) using unsterilized needles, and (4) sharing injection paraphernalia. To correct for multiple comparisons across these four outcome variables, a Bonferroni correction was performed and a reduced *α* of 0.0125 (0.05 ÷ 4) was used as the criterion for statistical significance (*p* values for all statistical tests are reported in Tables 3, 4, 5, 6). Each model consisted of two levels. The level-1 and level-2 model equations as well as the mixed model equation are detailed in Fig. 1. In the level-1 model the log count of the outcome measure was predicted by an intercept and the number of days since baseline assessments. In the level-2 model, the level-1 intercept was predicted by participant gender (male = 0; female = 1). The coefficient (linear trend) of time was predicted by an intercept, participant gender, treatment condition (buprenorphine initiated in the community = 0; buprenorphine initiated in prison = 1), community treatment entry (entered treatment within 10 days of release from prison: no = 0; yes = 1) and the interaction effect between treatment condition and community treatment entry. The intercept for the coefficient of time is the change in log counts of the outcome variable per day when all other predictors are equal to zero. This intercept determines the change in event rate ratio (ERR) for male participants assigned to begin buprenorphine in the community, but who failed to enter treatment. The treatment condition by community treatment interaction term was deleted from the final model if it was not statistically significant. Hierarchical models were fit to the data using HLM for Windows Version 7.03.

## Results

### Participants

Participants in the present study were 211 adults incarcerated in prison who met the criteria for DSM-IV defined opioid dependence at the time of incarceration and had between 3 and 9 months remaining before their anticipated release. The sample was 70.1% male (randomized to begin buprenorphine in prison *n* = 72; randomized to begin buprenorphine in the community *n* = 76) and 29.9% female (randomized to begin buprenorphine in prison *n* = 32; randomized to begin buprenorphine in the community *n* = 31). The majority of participants self-identified as Black (*n* = 148; 70.1%); the next largest group was Whites (*n* = 54; 25.6%) followed by American Indians (*n* = 3; 1.4%), Hispanics (*n* = 2; 0.9%), Asians and Pacific Islanders (*n* = 2; 0.9%), and those participants who identified as belonging to some other racial group (*n* = 2; 0.9%). On average, participants were 39.08 years of age (*SD* = 8.8). The average age of heroin use onset was 19.3 years of age (*SD* = 5.9) and the average age of participants when they were first incarcerated was 21.0 years old (*SD* = 7.5). Participants reported using heroin on 24.5 (*SD* = 10.1) of their last 30 days in the community on average. The vast majority of participants had received prior substance use treatment (*n* = 173; 81.9%) but only about one third (*n* = 67; 31.8%) had received prior methadone treatment and an even smaller proportion reported having received prior buprenorphine treatment (*n* = 32; 15.2%). Although participants were guaranteed entry into a community treatment program if they began treatment within 10 days of release (as part of the intervention) only 82 (38.9%; randomized to begin buprenorphine in prison *n* = 48; randomized to begin buprenorphine in the community *n* = 34) entered community treatment within 10 days of release. Table 1 summarizes the frequencies of predictor variables included in the statistical model by treatment condition.

**Table 1:** Frequencies of predictor variables included in the hierarchical over-dispersed Poisson models summarized by treatment condition

	Full sample *N* = 211	Buprenorphine in community *N* = 107	Buprenorphine in prison *N* = 104
Gender			
Male	148 (70.14%)	76 (71.03%)	72 (69.23%)
Female	63 (29.86%)	31 (28.97%)	32 (30.77%)
Entered community treatment			
Yes	82 (38.86%)	34 (31.78%)	48 (46.15%)
No	129 (61.14%)	73 (68.22%)	56 (53.85%)

### HIV-risk behaviors

The means and standard deviations of each outcome variable at baseline (study entry) and 12-month follow-up are presented in Table 2. Values for both the full sample and each treatment condition are provided at the earliest and latest time points in the study for comparison. Means displayed in the table take into account all available data at the given measurement point.

**Table 2:** Means (standard deviations) of counts of HIV-risk behaviors at baseline versus 12-month follow-up (*N* = 211)

	Full sample *N* = 211		Buprenorphine in community *N* = 107		Buprenorphine in prison *N* = 104	
	Baseline	12 months	Baseline	12 months	Baseline	12 months
Sex without a condom	22.97 (34.69)	12.18 (14.02)	21.42 (27.66)	11.88 (13.04)	24.57 (40.80)	12.46 (14.99)
Injection drug use	35.97 (63.66)	14.94 (48.68)	35.92 (67.81)	9.54 (27.92)	36.01 (59.47)	20.53 (63.09)
Using unsterilized needles	9.93 (30.28)	5.96 (20.58)	8.75 (26.20)	8.38 (27.37)	10.92 (33.55)	3.87 (12.83)
Sharing injection paraphernalia	26.16 (55.28)	11.07 (31.04)	35.08 (67.73)	14.08 (30.61)	19.10 (42.43)	8.47 (32.24)

### Sex without a condom

Results for the multilevel Poisson model are summarized in Table 3. There were no significant differences between genders in the frequency of having sex without a condom at baseline assessments [event rate ratio (*ERR*) = 1.09, 95% confidence interval (*95% CI*) = (0.79, 1.53)]. There was a significant effect of time such that for the reference group (male participants assigned to initiate buprenorphine in the community who failed to enter community treatment) the incidence of sex without a condom decreased by 3.6% for every 30 days in the community [*ERR* = 0.99, *95% CI* = (0.998, 0.999)]. There were no gender differences in the rate at which incidences of risky sex changed over time [*ERR* = 1.00, *95% CI* = (1.00, 1.00)]. Surprisingly, neither was there a significant effect of either treatment condition [*ERR* = 1.00, *95% CI* = (1.00, 1.00)] on the rate of change of risky sex behaviors, nor was there a significant relationship between entry into community treatment [*ERR* = 1.00, *95% CI* = (1.00, 1.00)] and the rate of change of risky sex behaviors.

**Table 3:** Results of the multilevel Poisson model for sex without a condom in the last 30 days

Effect	*b*	*SE*	*ERR (95% CI)*	*t*	*d.f.*	*p*
For intercept *π*_0_						
Intercept *β*_00_	2.70	0.09	14.88 (12.47, 17.75)	30.18	190	< 0.001
Gender *β*_01_	0.09	0.17	1.09 (0.79, 1.53)	0.54	190	0.59
For days slope *π*_1_						
Intercept *β*_10_	− 0.001	0.0003	0.99 (0.998, 0.999)	− 4.23	592	< 0.001
Condition *β*_11_	0.0007	0.0004	1.00 (1.00, 1.00)	1.90	592	0.059
Gender *β*_12_	0.0001	0.0004	1.00 (1.00, 1.00)	0.35	592	0.73
Community treatment entry *β*_13_	0.00002	0.0005	1.00 (1.00, 1.00)	0.04	592	0.97
Condition * community treatment entry *β*_14_	− 0.001	0.0007	0.99 (0.997, 1.00)	− 2.15	592	0.03

*N* = 211. *b* denotes the unstandardized partial regression coefficient; *SE* denotes its standard error. *ERR* denotes the event rate ratio for a one unit increase in the given variable. *t* denotes the *t* statistic for the given parameter estimate and *d.f.* is its degrees of freedom. Participant gender; male = 0; female = 1. Treatment condition; buprenorphine initiated in the community = 0; buprenorphine initiated in prison = 1. Whether or not the participant entered community treatment within 10 days of release from prison; no = 0; yes = 1

### Injection drug use

Results of the multilevel Poisson model are summarized in Table 4. There were no significant gender differences either in the frequency of injection drug use at baseline [*ERR* = 1.72, 95% CI = (0.91, 3.26)] or in the rate of change over time [*ERR* = 1.00, *95% CI* = (1.00, 1.00)]. There was a significant effect of time such that for the reference group (male participants assigned to initiate buprenorphine in the community who failed to enter community treatment) the incidence of injection drug use decreased by 6.7% for every 30 days in the community [*ERR* = 0.997, *95% CI* = (0.997, 0.998)]. There was a significant effect of treatment condition on the rate of change in injection drug use over time [*ERR* = 1.002, *95% CI* = (1.001, 1.003)]. Surprisingly, the group that was assigned to begin buprenorphine in the community was found to have a greater decline in injection drug use such that per 30 days since study entry their frequency of injection drug use was 4.7% less than that of the condition assigned to begin buprenorphine in prison. Lastly, there was no significant effect of community treatment entry [*ERR* = 0.999, *95% CI* = (0.998, 1.00)] on incidences of injection drug use.

**Table 4:** Results of the multilevel Poisson model for injection drug use in the last 30 days

Effect	*b*	*SE*	*ERR (95% CI)*	*t*	*d.f.*	*p*
For intercept *π*_0_						
Intercept *β*_00_	2.07	0.18	7.92 (5.57, 11.28)	11.22	200	< 0.001
Gender *β*_01_	0.55	0.32	1.72 (0.91, 3.26	1.69	200	0.09
For days slope *π*_1_						
Intercept *β*_10_	− 0.002	0.0004	0.997 (0.997, 0.998	− 6.26	744	< 0.001
Condition *β*_11_	0.002	0.0005	1.002 (1.001, 1.003)	3.42	744	< 0.001
Gender *β*_12_	− 0.0003	0.0005	1.00 (1.00, 1.00)	− 0.56	744	0.58
Community treatment entry *β*_13_	− 0.001	0.0006	0.999 (0.998, 1.00)	− 2.37	744	0.02
Condition * community treatment entry *β*_14_	–	–	–	–	–	–

*N* = 211. *b* denotes the unstandardized partial regression coefficient; *SE* denotes its standard error. *ERR* denotes the event rate ratio for a one unit increase in the given variable. *t* denotes the *t* statistic for the given parameter estimate and *d.f.* is its degrees of freedom. Participant gender; male = 0; female = 1. Treatment condition; buprenorphine initiated in the community = 0; buprenorphine initiated in prison = 1. Whether or not the participant entered community treatment within 10 days of release from prison; no = 0; yes = 1

### Using unsterilized needles

Results of the multilevel Poisson model are summarized in Table 5. There were no significant gender differences either in the frequency of using unsterilized needles at baseline [*ERR* = 1.74, *95% CI* = (0.57, 5.27)] or in the rate of change over time [*ERR* = 1.00, *95% CI* = (1.00, 1.00)]. Nor was there a main effect of time on the incidence of using unsterilized needles [*ERR* = 1.00, *95% CI* = (1.00, 1.00)]. There were also no significant effects of either treatment condition [*ERR* = 1.00, *95% CI* = (1.00, 1.00] or entering community treatment [*ERR* = 1.00, *95% CI* = (0.98, 1.00)] on the rate of change over time in instances of using unsterilized needles.

**Table 5:** Results of the multilevel Poisson model for using unsterilized needles in the last 30 days

Effect	*b*	*SE*	*ERR (95% CI)*	*t*	*d.f.*	*p*
For intercept *π*_0_						
Intercept *β*_00_	1.15	0.38	3.16 (1.50, 6.65)	3.02	94	0.003
Gender *β*_01_	0.55	0.56	1.74 (0.57, 5.27)	0.99	94	0.33
For days slope *π*_1_						
Intercept *β*_10_	− 0.002	0.002	1.00 (1.00, 1.00)	− 1.20	120	0.23
Condition *β*_11_	− 0.0003	0.002	1.00 (1.00, 1.00)	− 0.15	120	0.88
Gender *β*_12_	− 0.0002	0.002	1.00 (1.00, 1.00)	− 0.11	120	0.91
Community treatment entry *β*_13_	− 0.01	0.006	1.00 (0.98, 1.00)	− 1.58	120	0.12
Condition * community treatment entry *β*_14_	–	–	–	–	–	–

*N* = 211. *b* denotes the unstandardized partial regression coefficient; *SE* denotes its standard error. *ERR* denotes the Event Rate Ratio for a one unit increase in the given variable. *t* denotes the *t* statistic for the given parameter estimate and *d.f.* is its degrees of freedom. Participant gender; male = 0; female = 1. Treatment condition; buprenorphine initiated in the community = 0; buprenorphine initiated in prison = 1. Whether or not the participant entered community treatment within 10 days of release from prison; no = 0; yes = 1

### Sharing injection paraphernalia

Results of the multilevel Poisson model are summarized in Table 6. There were no significant gender differences either in the frequency of sharing injection paraphernalia at baseline [*ERR* = 1.38, *95% CI* = (0.60, 3.19)] or in the rate of change over time [*ERR* = 1.00, *95% CI* = (1.00, 1.00)]. However, there was a significant effect of time such that for the reference group (male participants assigned to initiate buprenorphine in the community who failed to enter community treatment) the incidence of sharing injection paraphernalia decreased by 12.1% for every 30 days in the community [*ERR* = 0.996, *95% CI* = (0.993, 0.998)]. There were also no significant effects for either treatment condition [*ERR* = 1.00, *95% CI* = (1.00, 1.00)] or entering community treatment [*ERR* = 1.00, *95% CI* = (0.98, 1.00)] on the rate of change over time in instances of sharing injection paraphernalia.

**Table 6:** Results of the multilevel Poisson model for sharing injection paraphernalia in the last 30 days

Effect	*b*	*SE*	*ERR (95% CI)*	*t*	*d.f.*	*p*
For intercept *π*_0_						
Intercept *β*_00_	2.51	0.28	12.30 (7.11, 21.30)	9.05	94	< 0.001
Gender *β*_01_	0.32	0.42	1.38 (0.60, 3.19)	0.77	94	0.45
For days slope *π*_1_						
Intercept *β*_10_	− 0.004	0.001	0.996 (0.993, 0.998)	− 3.4	118	< 0.001
Condition *β*_11_	0.003	0.002	1.00 (1.00, 1.00)	1.92	118	0.06
Gender *β*_12_	0.0003	0.002	1.00 (1.00, 1.00)	0.20	118	0.84
Community treatment entry *β*_13_	− 0.007	0.004	0.99 (0.99, 1.00)	− 1.72	118	0.09
Condition * community treatment entry *β*_14_	–	–	–	–	–	–

*N* = 211. *b* denotes the unstandardized partial regression coefficient; *SE* denotes its standard error. *ERR* denotes the event rate ratio for a one unit increase in the given variable. *t* denotes the *t* statistic for the given parameter estimate and *d.f.* is its degrees of freedom. Participant gender; male = 0; female = 1. Treatment condition; buprenorphine initiated in the community = 0; buprenorphine initiated in prison = 1. Whether or not the participant entered community treatment within 10 days of release from prison; no = 0; yes = 1

## Discussion

Overall, the present study did not find support for the initiation of buprenorphine in prison (as opposed to the community) as a means to reduce incidences of HIV-risk behaviors. When examining injection drug use, the condition assigned to begin buprenorphine in the community reported fewer instances of injecting over time when compared to the group assigned to initiate buprenorphine in prison. This result is in contrast to the parent study [49] that did not find any differences in self-reported heroin use, cocaine use, or treatment retention at 12 months. However, these results should be interpreted cautiously as only 40.3% of participants at baseline reported injecting drugs with a needle at least once in the last 30 days they were in the community suggesting they may prefer alternative routes of administration [60]. Therefore, changes in heroin or cocaine use over time would not necessarily mirror changes in injection drug use.

There were no significant predictors of decreasing incidences of sex without a condom, using unsterilized needles, or sharing injection paraphernalia in the present study. However, it should be noted that in the case of using unsterilized needles, self-reported frequencies of this behavior were very low at baseline compared to the other HIV-risk behaviors (see Table 2) as some individuals use opioids exclusively intranasally.

The present findings contribute to the existing efforts to reduce HIV-risk behavior among prisoners treated with OAT. We are aware of only one other randomized trial of pre-release buprenorphine treatment in the US, which was conducted among short-sentenced inmates in New York City [61]. That study did not report HIV risk behavior [61]. In a review of the research on prison-based OAT and its effects on HIV-risk behaviors, Larney found some support for the use OAT in prison to reduce post-release injection drug use and needle sharing [39]. However, the author noted a paucity of research in this area: only one of the five studies evaluated was a randomized controlled trial, and none of them took place in the US. Subsequent to the report by Larney, our group reported on post-release HIV-risk behavior from a randomized trial comparing initiating methadone treatment during vs. post-release from prison compared to a counseling in prison and referral condition [40]. That study did not find not find significant differences between treatment conditions in the rate of change of HIV-sex or -drug risk behavior. However there were significant effects of treatment condition on drug-risk behaviors such that participants assigned to initiate methadone pre-release reported fewer incidences of drug-risk behaviors irrespective of time and there was a significant effect of time such that participants reported fewer incidences of drug-risk behavior as the study progressed.

While there is limited research on the effects of prison-initiated OAT on HIV-risk behaviors, there is evidence supporting the use of community-based OAT to reduce HIV-behaviors for both criminal-justice-involved persons [26] and the general population [62]. A review of HIV-risk reduction strategies for criminal-justice-involved adults found that OAT significantly reduced injection drug use, but was less effective at reducing risky sexual behaviors [26]. These findings are echoed in reviews of studies involving general community samples [62]. Treatment adherence appears to be a significant component for the success of OAT in reducing HIV-risk behaviors [62], consistent with the broader literature which reports that adherence to substance abuse treatment is key in producing positive treatment outcomes [63]. While the present study did not find support for the use of pre-release buprenorphine to reduce HIV-risk behaviors, the parent study found that participants who initiated buprenorphine in prison had a higher mean number of days receiving buprenorphine treatment in the community, suggesting that prison-initiated buprenorphine may improve treatment adherence in the community.

The present study has a few important limitations. First, response rates to certain items were quite low at the most distal follow-up periods (6 and 12 months) compared to response rates at baseline and follow-ups closer to release and the overall rate of missing responses was high (54%). While multilevel modelling is equipped to handle missing data, if data are not missing completely at random [e.g., there is some systematic factor(s) accounting for missingness that is not included in the model], then information is lost in the analysis, potentially biasing parameter estimates. Second, an assumption of hierarchical linear models is a consistent effect of predictors over time, which may not be the case in reality when examining treatment effects that might be most potent in the time immediately after release. There is also an assumption of linear change over time when using such models. While model comparisons showed that quadratic and cubic functions did not appear to fit the data better than a linear one, changes in instances of a particular behavior may be asymptotic in reality as they have floors (e.g. counts of behaviors cannot be negative) and ceilings that cannot be exceeded due to real world constraints. Third, self-reported data on HIV risk is subject to potential bias although such bias would likely be present across both conditions. Finally, recall of these behaviors, particularly for the 30 days prior to index incarceration, is subject to potential inaccuracy, which again would likely be equally present across both conditions.

More research is needed on the intersection of prison-initiated pharmacotherapy and HIV treatment. At least one study [64] has found a positive association between 24-week retention in buprenorphine treatment and maximal viral suppression (which is associated with improved HIV treatment outcomes). However, this study was quasi-experimental, as participants in a RCT of directly administered antiretroviral therapy for prisoners who met the DSM-IV criteria for opioid dependence were offered buprenorphine pharmacotherapy while incarcerated and assessed post-release. Further research is needed to explore the relationship between prison-based OAT and HIV treatment outcomes such as viral suppression and medication adherence.

## Conclusions

Prison-initiated pharmacotherapy and continuing care in the community is still a promising intervention for improving treatment engagement in the community post-release, however, its merits as an intervention to reduce HIV-risk behaviors have not yet been demonstrated. The wealth of research on the nexus of substance use and HIV risk suggests that reductions in substance use appear to be an important precursor to subsequent reductions in HIV-risk behavior. Therefore, future efforts to reduce HIV-risk behaviors among people with substance use disorders should focus on implementing the ‘gold standard’ of evidence-based treatment, medication-assisted treatment, and focus on effectively engaging patients in that treatment.

## Data Availability

The datasets used and analyzed during the current study are available from the corresponding author upon reasonable request.

## Abbreviations

OUD: opioid use disorder
OAT: opioid agonist therapy
ERR: event rate ratio
CI: confidence interval
